# Supplementary material for: Identification of photoperiod-induced specific miRNAs in the adrenal glands of Sunite sheep (Ovis aries)
Source: Front Vet Sci. 2022 Jul 22;9:888207. doi: 10.3389/fvets.2022.888207 (PMC9354845; doi:10.3389/fvets.2022.888207)
Supplement: Supplementary file 1 [file Data_Sheet_1.ZIP › Supplementary file/miRDeep2 file.zip!/miRDeep2 file/Novel_Structure/LP42A2/Novel_367.pdf]

Provisional ID	:	NC_019484.2_37851
Score total	:	25
Score for star read(s)	:	3.9
Score for read counts	:	17.5
Score for mfe	:	2
Score for randfold	:	1.6
Score for cons. seed	:	
Total read count	:	46
Mature read count	:	45
Loop read count	:	0
Star read count	:	1

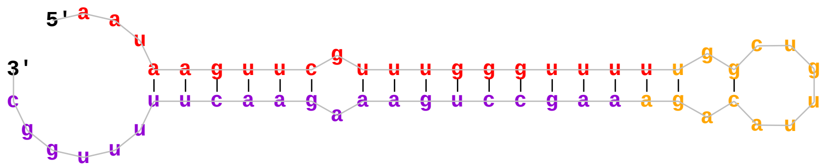

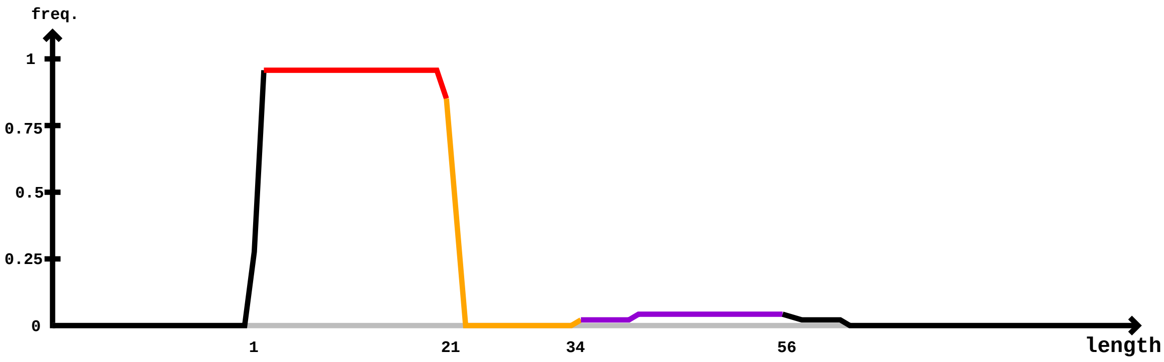

Mature Star

```
5'- uggauauauaugggguuggccaauaaguucguuuggguuuuuggcuguuacagaaagccugaaagaacuuuuuggccaacccaauauauuucaaauaucuuuuuccuuuucu   -3'    obs
    uggauauauauggguuggccaauaaguucguuuggguuuuuggcuguuacagaaagccugaaagaacuuuuuggccaacccaauauauuucaaauaucuuuuuccuuuucu          exp
    .((((((((.((((((((((((.((((((.(((((((((((.(......).))))))))))).)))))).))))))))))))))))))))).....................    reads    mm    sample
    ...................caaAaaguucguuuggguuuuu......................................................................    13       1     seq
    ....................aaAaaguucguuuggguuu........................................................................    5        1     seq
    ....................aaAaaguucguuuggguuuu.......................................................................    24       1     seq
    ....................aauaaguucguuuggguuuuu......................................................................    3        0     seq
    .....................................................aagccugaaUgaacuuuuuggc....................................    1        1     seq
    ...........................................................gaaagaacAuuuuggccaaccc..............................    1        1     seq
```
